# Clinical Spectrum and Neuroimagistic Features in Hospitalized Patients with Neurological Disorders and Concomitant Coronavirus-19 Infection

**DOI:** 10.3390/brainsci11091138

**Published:** 2021-08-27

**Authors:** Anca Elena Gogu, Andrei Gheorghe Motoc, Alina Zorina Stroe, Any Docu Axelerad, Daniel Docu Axelerad, Florina Pârv, Georgiana Munteanu, Flavius Dan, Dragos Catalin Jianu

**Affiliations:** 1 Department of Neurology, “Victor Babeș” University of Medicine and Pharmacy, 300041 Timișoara, Romania; anca.gogu@umft.ro (A.E.G.); isfan_georgiana@yahoo.com (G.M.); danflavius88@yahoo.com (F.D.); dcjianu@yahoo.com (D.C.J.); 2 Centre for Cognitive Research in Neuropsychiatric Pathology (NeuroPsy-Cog), “Victor Babeș” University of Medicine and Pharmacy, 300041 Timișoara, Romania; amotoc@umft.ro; 3 Department of Anatomy and Embryology, “Victor Babeș” University of Medicine and Pharmacy, 300041 Timișoara, Romania; 4 Department of Neurology, General Medicine Faculty, “Ovidius” University, 900470 Constanta, Romania; docuaxi@yahoo.com; 5 Brainaxy Clinic, 900628 Constanta, Romania; docuaxy@yahoo.com; 6 Department of Cardiology, “Victor Babeș” University of Medicine and Pharmacy, 300041 Timișoara, Romania; florina_parv@yahoo.com

**Keywords:** COVID-19 infection, neurological disorders, stroke

## Abstract

In the first months of the COVID-19 pandemic, several research studies focused on understanding the damage to the respiratory and circulatory systems. However, the evidence of neurological manifestations as part of the clinical spectrum of the disease has increased. The aim of this retrospective study was to determine the potential association of neurological disorders with concomitant COVID-19 infection. We reviewed 101 patients (mean age, 70.05 years; 62.37% men) diagnosed with different neurological disorders and COVID-19 who were referred to the Department of Neurology between March 2020 and May 2021. The protocol included demographic, clinical, and neuroimagistic features, biochemical evaluation data, and prognosis. In the first group of patients with non-severe COVID-19 infection (<50% lung damage), we enrolled 75 cases (mean age, 69.13 years; 65.33% men), and the second group, with 26 patients (mean age, 72.69 years; 53.84% men), developed severe COVID-19 infection (>50% lung damage). Severe COVID-19 infection was significantly correlated with an increased highly sensitive C-reactive protein level (hsCRP) (*p* < 0.05), lactate dehydrogenase level (LDH) (*p* < 0.05), erythrocyte sedimentation rate (ESR) (*p* < 0.05), D-dimer (*p* < 0.05), fibrinogen level (*p* < 0.05), and blood glucose (*p* < 0.05) when compared to the first group. These biochemical parameters were increased in both groups, but the levels were much higher in the second group. Headaches (72.27%) and dizziness (14.85%) were present in the early stage of infection. Cerebrovascular events were also reported: ischemic stroke (48% vs. 57.69%; *p* < 0.05), cerebral hemorrhage (4.95%), and cerebral venous sinus thrombosis (1.98%). Encephalitis (1.98%) and Guillain–Barré Syndrome (1.98%) were found but less frequently. Cranial nerve abnormalities were statistically more common in the non-severe group: anosmia (32% vs. 26.92%; *p* < 0.05), dysgeusia/ageusia (48% vs. 42.30%; *p* < 0.05), impaired eye movement (1.33% vs. 0%), and facial nerve palsy (2.66% vs. 0%). Seizures (13.33% vs. 11.53%; *p* < 0.05) and a depressed level of consciousness (31.68%) occurred commonly. We detected the neuropsychiatric symptoms of anxiety (23.76%) and depression (14.85%). Mortality was increased in both groups but was much higher in the second group (46.15% vs. 21.33%). Neurological complications during COVID-19 infection are common in hospitalized patients, but the mechanism of these complications is not fully understood, representing a continuous challenge for neurologists.

## 1. Introduction

Several research studies have been performed, focusing on the understanding of acute respiratory syndrome and treatment in patients with COVID-19 infection in the first months of the pandemic. However, evidence of neurological manifestations as part of the clinical spectrum of the disease has increased. Similarly, the other coronavirus (CoV) epidemics, such as severe acute respiratory syndrome (SARS-CoV-1) in 2003 and Middle East respiratory syndrome (MERS-CoV) in 2012, have been associated with neurological complications [1,2,3]. The pathophysiology of COVID-19 in the central nervous system shows that COVID-19 has a selective tropism for neurons and can use the axonal transport system as a means of neuron-to-neuron propagation, and they are also capable of inducing acute and persistent infection in neurons, oligodendrocytes, and neuroglia [4,5]. COVID-19 is able to enter the central nervous system through the olfactory nerves, following its path toward the thalamus and brainstem. A higher inoculum has been found in the brainstem and brain compared to the lung tissue, which shows that the central nervous system is affected more rapidly and frequently than the lungs and has greater implications in terms of gravity and the mortality rate [6,7,8,9]. Herein, we report a study of 101 SARS-CoV-2-positive patients presenting with neurological symptoms as the initial manifestation of the infection.

## 2. Materials and Methods

### 2.1. Patient Population

This was a retrospective study including 101 patients (63 males and 38 females) with neurological disorders and concomitant COVID-19 infection referred to the Neurology Department of Timisoara County Emergency Clinical Hospital between March 2020 and May 2021 among 1866 total cases admissions, which represents 5.41%. We reviewed the medical records of all patients admitted with neurological disorders and concomitant SARS-CoV-2 RNA infection detected by real-time reverse transcription-polymerase chain reaction (RT-PCR). Demographic (sex and age), clinical, radiologic examination (head CT, brain MRI, and chest CT), laboratory parameters (leucocyte, lymphocyte, and thrombocyte count; blood glucose, lactate dehydrogenase (LDH), highly sensitive C-reactive protein (hsCRP); erythrocyte sedimentation rate (ESR); D-dimer; fibrinogen; activated partial thromboplastin time (APTT); prothrombin time (PT); and international normalized ratio (INR)); and lumbar puncture and neurophysiologic test (EEG and EMG) data, if indicated, were all considered [10].

We classified the patient population into two groups. In the first group, we included 75 patients with different neurological disorders and non-severe COVID-19 infections, meaning <50% lung damage. In the second group, we enrolled 26 patients, recruited from the Neurology Department, with severe COVID-19 infections (>50% lung damage). The severity of COVID-19 was defined according to the 2007 Infectious Diseases Society of America/American Thoracic Society Criteria [11]. Included in the study were patients with comorbidities such as hypertension, type II diabetes mellitus, dyslipidemia, atrial fibrillation, and previous neurological comorbidities such as prior stroke, Myasthenia Gravis, and Parkinson’s Disease. The exclusion criteria were head trauma, brain tumors, oncologic pathologies, and psychiatric disorders [12].

This retrospective and noninterventional study was approved by the Ethics Committee for Clinical Studies of the Timisoara County Emergency Clinical Hospital (registration number 259/14.07.2021) and was conducted in accordance with the Declaration of Helsinki. We obtained written informed consent for enrollment from all participants.

### 2.2. Clinical and Biochemical Evaluation

Evaluation of the general and neurological clinical conditions was conducted for all patients on admission by the neurologist. The research group categorized the neurologic manifestations into nonspecific symptoms (headaches and dizziness), cerebrovascular events (ischemic stroke, cerebral hemorrhage, cerebral venous sinus thrombosis, and seizures), inflammatory manifestations (encephalitis and Guillain–Barré Syndrome), cranial nerve abnormalities (anosmia, dysgeusia/ageusia, impaired eye movement, and facial nerve palsy); depressed level of consciousness (acute confusional syndrome and coma); and neuropsychiatric symptoms (anxiety and depression). We included patients with prior stroke, Myasthenia Gravis, and Parkinson’s Disease who presented with an altered neurological status concomitant with COVID-19 infection.

Blood samples were collected for each patient on admission, and the results were provided by the medical laboratory of our hospital. The blood test parameters were documented, with the following normal reference values: Leucocyte count, 4–9.5 × 1000/L; lymphocyte count, 0.8–3.8 × 1000/L; thrombocyte count, 150–400 × 1000/L; blood glucose, 65–105 mg/dL; LDH, 120–246 U/L; hsCRP, 0–10 mg/L; ESR, 1–15 mm/1 h; D-dimer, 0–243 ng/mL; fibrinogen, 200–393 mg/dL; APTT, 25.1–36.5 s; PT, 9.4–12.5 s; and INR, 0.80–1.07 INR.

### 2.3. Radiologic Findings and Electrophysiological Studies of Nerve Conduction

The lung involvement was documented by chest-computed tomography (chest CT) for every patient on admission to the emergency room. The results were interpreted by radiologists at the Timisoara County Emergency Clinical Hospital. They divided patients according to the degree of lung damage, unilaterally or bilaterally, considering pneumonia, bronchopneumonia, and pleurisy caused by SARS-CoV-2 infection. In the first group of patients with the non-severe form were those with less than 50% lung damage, while in the second group of patients with the severe form of the disease were those with more than 50% lung damage.

Non-contrast head-computed tomography (head CT) was performed for patients on admission to hospital. The accessibility of this test, which obtains data on brain damage, allowed us to use it for all patients admitted to the Neurology Clinic. Imagistic evaluation by magnetic resonance imaging (MRI) was performed for every patient between the first 48 h of admission. MRI sequences were performed with a 1.5-T MR unit (Siemens Medical System, Erlangen, Germany), including T1- and T2-weighted sequences, fluid-attenuated inversion recovery (FLAIR) imaging sequence, diffusion-weighted imaging (DWI) with apparent diffusion coefficient (ADC) calculations, and susceptibility-weighted imaging (SWI) [13]. MR angiography (three-dimensional time-of-flight 3D-TOF sequences) and MR venography (2D-TOF) were considered the gold standard in diagnosing cerebral venous sinus thrombosis [14].

Concerning COVID-19, in two patients were described different variants of Guillain–Barré Syndrome (GBS) with motor deficits (tetraplegia) and sensory disturbances on the clinical neurologic examination. Patients were investigated with nerve conduction studies (NCSs) and had abnormal findings on sensory NCSs. Moreover, in those patients that underwent lumbar puncture were abnormalities of the cerebrospinal fluid (CSF), such as albumincytological dissociation.

### 2.4. Statistical Analyses

Statistical calculations were performed using Microsoft Excel (Microsoft Corporation, Albuquerque, NM, USA, version 2019).

The chi-square test was used for testing hypotheses with nominal variables by comparing the observed data values to the expected values.

The Fisher’s least significant difference (LSD) procedure was applied as a two-step testing procedure for pairwise comparisons of several simple-sized data groups, such as the gender variables. The null hypothesis in this context was computed by considering all the possible values that could provide the tables observed. For some outcomes, such as for the symptoms used as the input in the second table, the traditional *p*-value based on the approximated Student’s distribution substantially succeeded the minimum attainable Fisher’s exact *p*-value. For other outcomes, such as pneumonia or gender comparison the Fisher’s exact null randomization distribution substantially differed from the bell-shaped one assumed by the Z-test.

The Student’s *t*-test was used in the second table for the majority of variables employed as the input based on different combinations of data: independent samples *t*-test, paired samples *t*-test, and one-sample *t*-test, where the variance was not assumed to be equal.

A *p*-value, or probability value, is a number computed by statistical software that describes how likely the used data would have occurred by random chance (i.e., that the null hypothesis is true). A smaller *p*-value means that there is stronger evidence in favor of the alternative hypothesis. Therefore, a *p*-value < 0.05 was used for significant differences. Examples of interpretation of the *p*-value can be seen in Section 3.1. 

Wilcoxon–Mann–Whitney tests were applied for the hypothesis testing and statistical modeling to assess the distribution of the observations obtained between groups.

## 3. Results

### 3.1. Demographic Characteristics

A total of 101 patients with different neurological diseases and a confirmed concomitant SARS-CoV-2 infection by RT-PCR on admission to the Neurology Department of our hospital between March 2020 and May 2021 were recruited for this study. The mean age of the patients was 70.05 years (standard deviation (Std) = 11.21). There were 63 males (62.37%) and 38 females (37.63%). 

In the first group of patients with non-severe COVID-19 infection (<50% lung damage), we enrolled 75 cases (mean age, 69.13 years; Std = 11.64), 49 of whom were male (65.33%) and 26 female (34.67%). The second group comprised 26 patients (mean age, 72.69 years; Std = 9.55) who developed a severe COVID-19 infection (>50% lung damage), 14 of whom were male (53.84%) and 12 female (46.15%). There were significant differences (*p* < 0.05) between the two groups of patients in terms of age and gender distribution. 

The patients’ characteristics, including demographic, clinical, radiological, and biochemical data, are summarized in Table 1.

### 3.2. Clinical Features

The clinical spectrum of neurological disorders concomitant with COVID-19 infection at admission varied according to the disease etiology, age, time interval between the onset of disease and clinical presentation, and neurological comorbidities. The comorbidities were arterial hypertension—68 cases (67.32%), with 52 non-severe cases (69.33%) and 16 severe cases (61.53%); diabetes mellitus—26 cases (25.74%), with 18 non-severe cases (24%) and eight severe cases (30.76%); dyslipidemia—44 cases (43.56%), with 35 non-severe cases (46.66%) and 11 severe cases (42.30%); and atrial fibrillation—19 cases (18.81%) with 10 non-severe cases (13.33%) and nine severe cases (34.61%). Thirteen patients (12.87%) were identified has having preexisting neurological disorders: Eight cases (7.92%) with prior stroke, three cases (2.97%) with Myasthenia Gravis, and two cases (1.98%) with Parkinson’s Disease and COVID-19 infection. All of them developed exacerbated neurological symptoms and a severe form of COVID-19 with high mortality (Table 2).

The most common features were fever, dyspnea, and fatigue related with acute respiratory syndrome SARS-CoV-2 infection. There were significant differences (*p* < 0.05) between the two groups: 46 cases of fever (61.33%) in the first group vs. 24 cases (92.30%) in the second group, 51 cases of dyspnea (68%) in the first group vs. 24 cases (92.30%) in the second group, and 40 cases of fatigue (53.33%) in the first group vs. 22 cases (84.61%) in the second group of patients.

The most common nonspecific neurological symptoms of COVID-19 were headaches and dizziness. Headaches were reported in 55 patients (73.33%) from the first group with non-severe symptoms and in 18 patients (69.23%) from the second group; there was no significant difference between the two groups (*p* = 0.24). The severity of the headaches was reported to be moderate-to-severe with a tension-type quality [15]. Dizziness was reported in nine cases (12%) from the first group and in six cases (23.07%) from the second group of patients, without a significant difference between the two groups.

The cerebrovascular events reported in our study include acute ischemic stroke (51 cases; 50.49%), cerebral hemorrhage (five cases; 4.95%), and cerebral venous sinus thrombosis (two cases; 1.98%). Acute ischemic stroke was reported in adult patients with SARS-CoV-2 infection and with several comorbidities such as hypertension, diabetes mellitus, dyslipidemia, heart disease, and obesity. In the first group, we found 36 cases (48%) with acute ischemic stroke and 15 cases (57.69%) in the second group with the severe form of the disease (*p* < 0.05). Moreover, we included patients with prior stroke—five cases (6.66%) in the first group vs. three cases (11.53%) in the second group, with an exacerbation of neurological symptoms and high mortality. 

Cerebral hemorrhage was seen in five patients (4.95%)—two cases (2.66%) in the first group and three cases (11.53%) in the second group of patients. In critically ill patients, SARS-CoV-2 infection was associated with coagulopathies such as thrombocytopenia, elevated D-dimer, and a prolonged prothrombin time, which can result in hemorrhage [16]. SARS-CoV-2-induced ACE2 downregulation may lead to vasoconstriction and blood pressure spikes, which can cause arterial wall rupture and hemorrhage. All patients with cerebral hemorrhage had a very low level of cholesterol, which is an additional risk factor for intracranial hemorrhage [17].

Cerebral venous sinus thrombosis was reported in two patients (1.98%), one in the non-severe group and one in the second group with the severe form of the disease. Both had thrombosis of the superior sagittal and lateral sinuses, with parenchymal lesions such as hemorrhagic infarction at the frontoparietal lobe, related to the topography of the cerebral sinus thrombosis [18]. These young patients were hospitalized with superficial comas, generalized seizures, and motor deficits. We mentioned that the patients had inherited thrombophilia with the MTHFR C677T gene polymorphism–homozygous phenotype, but they also had a significant increase in D-dimers, high fibrinogen and hsCRP levels, and a mildly prolonged prothrombin time. A cytokine storm in severely ill COVID-19 patients is another possible mechanism, because it suppresses the anticoagulant pathways and releases the von Willebrand factor, which might lead to thrombosis in these patients [19].

**Figure 1:**
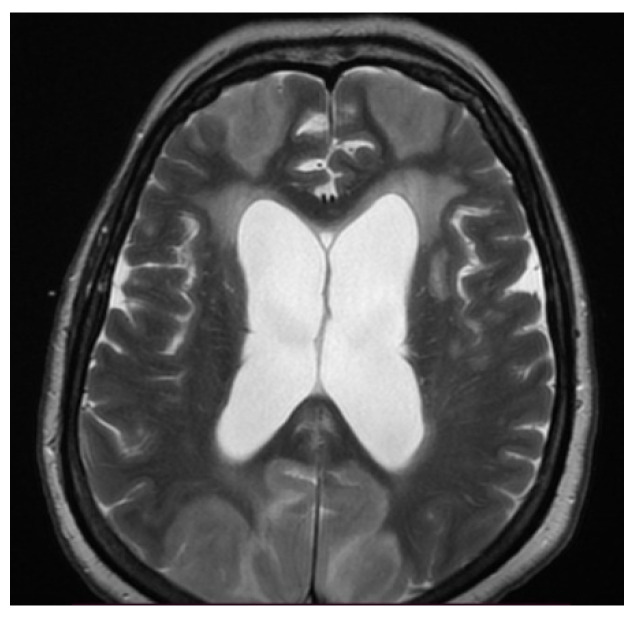
MRI axial T2-weighted sequence showed bilateral subcortical confluent hyperintense lesions with vasogenic edema in the frontal, parietal, and occipital lobes and secondary hydrocephalus.

**Figure 2:**
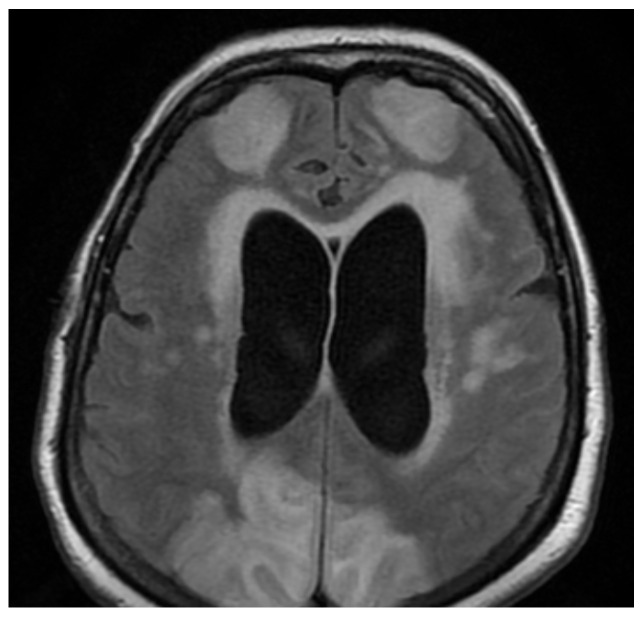
MRI axial fluid-attenuated inversion recovery (FLAIR) sequence noting bilateral subcortical hyperintense lesions, especially in the occipitoparietal lobes, resembling posterior reversible encephalopathy syndrome (PRES)-like findings.

**Figure 3:**
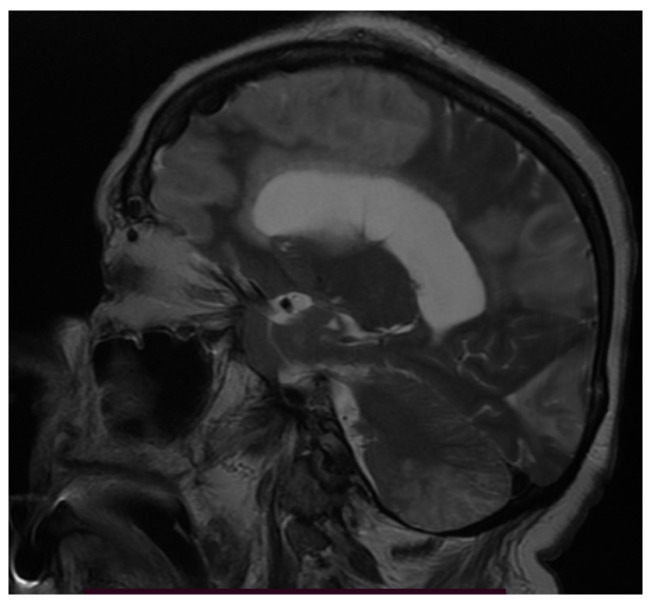
MRI sagittal T2-weighted sequence showing diffuse confluent hyperintensities in the peri-corpus callosum.

**Figure 4:**
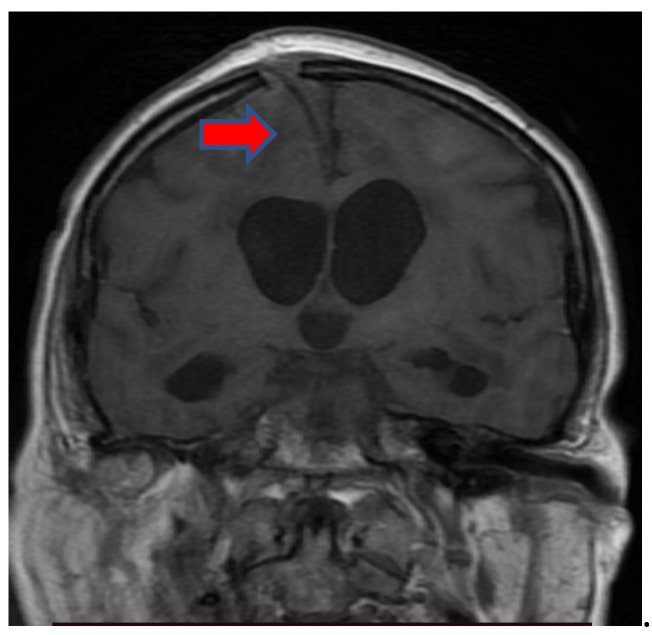
MRI coronal T1-weighted sequence noting secondary hydrocephalus that appeared as a complication of encephalitis, for which external drainage was installed (red arrow).

**Figure 5:**
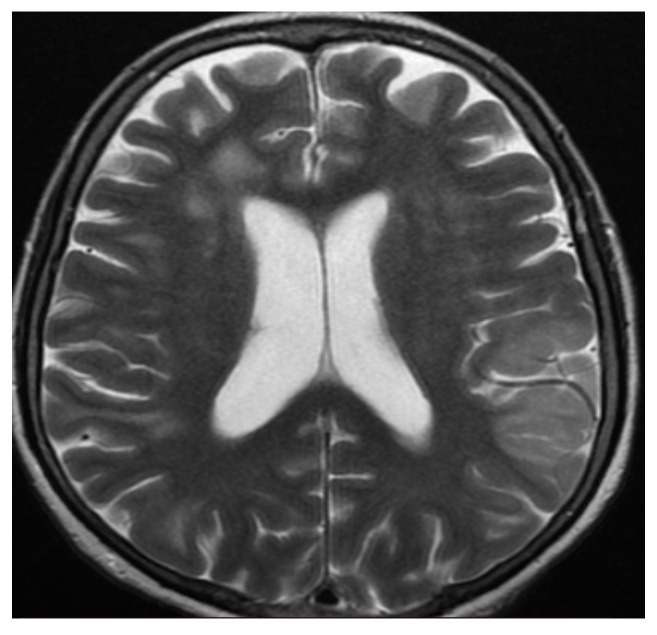
MRI axial T2-weighted sequence revealing bilateral diffuse confluent white matter hyperintensities in the frontal, parietal, and occipital lobes.

**Figure 6:**
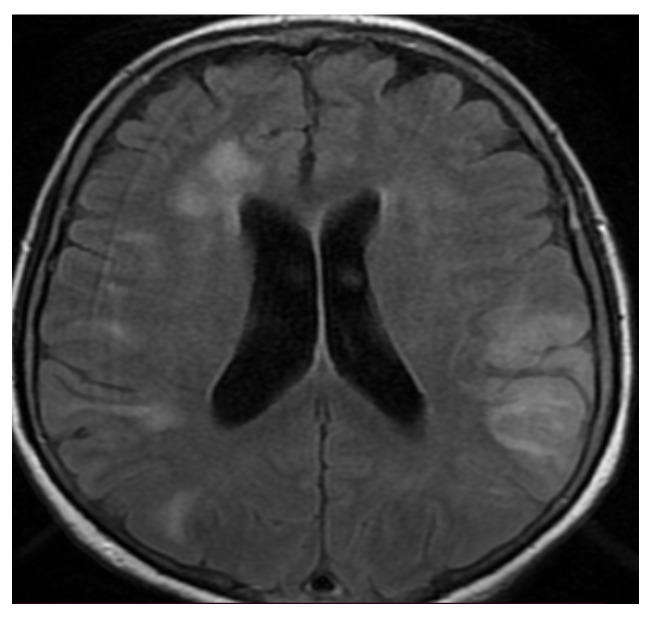
MRI axial fluid-attenuated inversion recovery (FLAIR) image showing bilateral subcortical hyperintense lesions in the frontal, parietal, and temporal lobes, without significant enhancement, compatible with encephalitis. MR venography 2D-TOF (2D time-of-flight) excluded cerebral venous sinus thrombosis.

When considering inflammatory manifestations, we detected two patients with encephalitis (1.98%) and two cases with Guillain–Barré Syndrome (GBS) (1.98%). Both patients with encephalitis were females, 78 and 76 years of age with comorbidities (hypertension, atrial fibrillation, and prior lacunar ischemic stroke), who were admitted to the intensive care unit due to severe bilateral pneumonia (RT-PCR-positive for severe acute respiratory syndrome coronavirus type 2) requiring mechanical ventilation. There, patients were in a superficial coma at onset, with a focal neurological deficit such as hemiparesis or paraparesis, psychomotor agitation, and generalized seizures. Neuroimaging (MRI) showed bilateral cortico-subcortical hyperintense lesions (Figure 1, Figure 2, Figure 3, Figure 5 and Figure 6) with vasogenic edema in the frontal, parietal, and temporal lobes. In one of the patients, secondary hydrocephalus appeared as a complication, for which external drainage was installed (Figure 4). In the first case (female, 78 years of age), the CSF analysis was negative for bacterial or fungal infections; the protein levels (10.09 g/L) and leucocyte number (50 uL) were increased in the CSF, although the glucose level was decreased (27 mg/dL). In the second case (female, 76 years of age), the CSF protein level was 7.2 g/L, the leucocyte number was 50 uL, and the glucose level was 50 mg/dL. The blood test parameters showed high levels of the blood glucose, LDH, hsCRP, ESR, D-dimer, fibrinogen, and severe thrombocytopenia. The blood cultures of the two patients were negative for aerobic germs, anaerobes, and fungi.

Guillain–Barré Syndrome (GBS) was reported at two patients (1.98) with the severe form of COVID-19 infection. The antibodies produced by the host immune system to fight the virus cross-reacted and bound to the peripheral nerves, causing neuronal damage [20]. These patients had motor deficits (tetraplegia) and sensory disturbances during their neurological examinations. Nerve conduction studies (NCSs) and abnormalities of the CSF, such as albumincytological dissociation, support the diagnosis.

Seizures were reported in 13 patients (12.87%)—10 cases (13.33%) in the first group and three cases (11.53%) in the second group, with a significant difference (*p* < 0.05). None of them were known to have epilepsy, and a CT scan of the head revealed no lesions. Three of the patients were complicated with status epilepticus; they had the severe form of COVID-19 infection, associated with older age and higher hsCRP and D-dimer levels. A computed tomography scan of the chest; a positive RT-PCR for SARS-CoV-2 infection; blood count; and inflammatory tests (hsCRP, D-dimer, and fibrinogen) advocate for the viral-infectious etiology of seizures. We think that the accumulation of inflammatory markers associated with COVID-19 infection and direct invasion of the virus into the brain may have caused the seizures. Hypoxia due to lung damage, sepsis, and metabolic disorders (hyponatremia and hypokalemia being present in six of the 13 patients) would be a trigger/aggravating factor of seizures.

Cranial nerve abnormalities were found in a lot of patients. Anosmia was reported in 31 cases (30.69%)—24 patients (32%) in the first group and seven patients (26.92%) in the severe group, with a significant difference between the two groups (*p* < 0.05). Anosmia was not related to the severity of the infection. Ageusia/dysgeusia was found in 47 cases (46.53%)—36 patients (48%) in the non-severe group and 11 patients (42.30%) in the severe group (*p* < 0.05). Impaired eye movement associated with COVID-19 was described only in one patient (0.99%) with the non-severe form of the disease. The patient had left abducens nerve palsy with a pontine lesion on their MRI. Facial nerve palsy was found in two patients (1.98%), both of them with the non-severe form of the disease. 

A depressed level of consciousness was observed in 32 patients (31.68%). Of note was that acute confusional syndrome and bradypsychia occurred in the context of hypoxemia in 25 cases (24.75%)—20 patients (26.66%) in the first group and five patients (19.23%) in the second group. A coma at onset was reported in seven patients (6.93%) with the severe form of the disease. In COVID-19 patients, possible mechanisms include hypoxemia, infections, inflammation, electrolyte imbalance, and toxic and metabolic encephalopathies [21]. The patients with orotracheal intubation at admission (six cases; 5.94%) with ventilatory mechanical assistance died, all of whom were female.

Neuropsychiatric symptoms were found in 39 cases (38.61%), anxiety being the most frequent symptom (24 cases; 23.76%). In the first group of non-severe patients, we found 18 cases (24%) with anxiety, with only six cases (23.07%) in the second group. Depression was detected by a psychiatrist in 15 cases (14.85%)—11 cases (14.66%) in the first group and four cases (15.38%) in the second group.

Patients with preexisting neurological disorders and COVID-19 developed more severe clinical symptoms and worse outcomes. Patients with myasthenia gravis (three cases; 2.97%) treated, according to the protocol, with human immunoglobulins and/or plasmapheresis died.

### 3.3. Neuroimaging

A native cerebral-computed tomography (cerebral CT) scan was conducted for every patient on admission to the hospital. The cerebral CT scan was normal in 28 patients (27.72%); a CT was useful for showing infarction, hemorrhagic infarction, subarachnoid hemorrhage, intraparenchymal hemorrhage, and hydrocephalus in 73 patients (72.27%). In the first 48 h after admission, we performed a MRI, including T1- and T2-weighted sequences, FLAIR imaging, and DWI and SWI sequences for all the patients. MR angiography (3D-TOF) and MR venography (2D-TOF) were obtained during the same imaging session for patients with cerebral venous sinus thrombosis. The results were interpreted by a radiologist at the Timisoara County Emergencies Clinical Hospital.

We next describe the most special cases with encephalitis, having various clinical neurological symptoms and different neuroradiological features.

The first case, a 78-year-old female, was hospitalized in the intensive care unit, requiring mechanical ventilation. She presented severe acute respiratory syndrome coronavirus type 2, with bilateral pneumonia, coma at onset, tetraparesis through double hemiparesis (right first and left after), and two generalized seizures. The imagistic features can be seen in Figure 1, Figure 2, Figure 3 and Figure 4.

The second case, a 76-year-old female, presented severe psychomotor agitation, paraparesis, meningeal symptoms, and bilateral severe pneumonia concomitant COVID-19 infection. The imagistic features can be seen in Figure 5 and Figure 6.

### 3.4. Biochemical Findings

Paraclinical blood-related investigations were performed for each patient on admission to the hospital. We selected, for our study, the most important blood test parameters in the context of SARS-CoV-2 infection in neurological patients: leucocyte count, lymphocyte count, thrombocyte count, blood glucose, LDH, hsCRP, ESR, D-dimer, fibrinogen, APTT, PT, and INR. We detected the leucocyte count (mean value = 12.85 × 1000/L; Std = 7.7), the lymphocyte count (mean value = 2.45 × 1000/L; Std = 5.41), the thrombocyte count (mean value = 233.53 × 1000/L; Std = 114.33), blood glucose (mean value = 148.05 mg/dL; Std = 88.01), LDH (mean value = 438.95 U/L; Std = 482.33), D-dimer (mean value = 1252.09 ng/mL; Std = 2115.51), fibrinogen (mean value = 535.02 mg/dL; Std = 189.41), APTT (mean value = 28.04 s; Std = 7.09), PT (mean value = 15.13 s; Std = 17.79), and INR (mean value = 1.49; Std = 1.62). In the studied patients (*n* = 101), we observed elevated leucocyte; blood glucose; LDH; PT; INR; and inflammatory markers (hsCRP, ESR, D-dimer, and fibrinogen) values. The increased values of these blood parameters are associated with the severe form of the disease.

We studied two groups of patients. By comparing patients from the first group with non-severe COVID-19 infection (<50% lung damage) vs. the second group of patients with the severe form of the disease (>50% lung damage), we detailed the leucocyte count (13 ± 8.27 × 1000/L vs. 12.42 ± 5.88 × 1000/L; *p* = 0.639), the lymphocyte count (2.78 ± 6.21 × 1000/L vs. 1.5 ± 1.23 × 1000/L; *p* = 0.288), the thrombocyte count (235.35 ± 118.54 × 1000/L vs. 228.26 ± 103.25 × 1000/L; *p* = 0.053), blood glucose (136.67 ± 61.66 mg/dL vs. 180.88 ± 134.99 mg/dL; *p* < 0.05), LDH (356.11 ± 290.39 U/L vs. 677.92 ± 775.23 U/L; *p* < 0.05), hsCRP (77.98 ± 95.4 mg/L vs. 116.42 ± 81.69 mg/L; *p* < 0.05), ESR (35.76 ± 16.67 mm/h vs. 51.5 ± 23.55 mm/h; *p* < 0.05), D-dimers (1206.4 ± 2250.4 ng/mL vs. 1383.88 ± 1699.08 ng/mL; *p* < 0.05), and fibrinogen (521.4 ± 178.94 mg/dL vs. 574.31 ± 215.78 mg/dL; *p* < 0.05). There were no significant differences between groups regarding APTT (27.62 ± 5.25 s vs. 29.24 ± 10.83 s; *p* = 0.471), PT (13.17 ± 2.84 s vs. 20.8 ± 34.59 s; *p* = 0.08), and INR (1.28 ± 0.28 vs. 2.53 ± 3.69; *p* = 0.421). The results showed statistically significant increased values for the leucocytes, blood glucose, LDH, hsCRP, ESR, D-dimer, fibrinogen, PT, and INR in both groups, but they were much higher in the second group. On the contrary, we observed statistically significant differences between groups (*p* < 0.05) regarding blood glucose, LDH, hsCRP, ESR, D-dimer, and fibrinogen.

## 4. Discussion

Reports on neurological complications during COVID-19 are growing on a daily basis. More and more scientific data suggest that severe acute respiratory syndrome coronavirus 2 (SARS-CoV-2) also involves the brain [22]. In our study, we tried to understand the impact of this novel virus on different neurological diseases. We were unable to demonstrate direct CNS invasion by SARS-CoV-2 in our study population who developed neurologic manifestations. In addition to direct CNS infection, the intense inflammatory response caused by SARS-CoV-2 infection can lead to blood–brain barrier (BBB) breakdown [23]. One mechanism described in the literature is an increased BBB permeability, which could make possible the circulation of peripheral cytokines through the CNS, and an indirect neuroinflammatory reaction could be responsible for neurologic manifestation in COVID-19 infection [23]. Two potential routes of virus entry in the brain are suggested: The first possible route is through the trigeminal and olfactory nerve endings. Infiltration through the olfactory tract would explain the lesion in the medial temporal lobe, while the lesions in the brainstem and thalamus could be categorized as a central infiltration through the trigeminal tract [24]. The second mechanism of viral invasion could be an increased permeability of the blood–brain barrier (BBB) through the high levels of proinflammatory cytokines in the cerebrospinal fluid [25].

After analyzing the distinctive demographic characteristics by gender, a prevalence of neurological manifestations emerged in male patients (62.37% vs. 37.63%; *p* < 0.05). By comparing the age and gender between the two groups of patients, we found a higher prevalence of older cases and female patients in the second group with the severe form of COVID-19 infection (mean age, 69.13 years vs. 72.69 years; female, 34.67% vs. 46.15%; *p* < 0.05).

The symptomatology was complex in relation to the etiology of the neurological disease. Headaches were the most common symptom (72.27%), followed by dysgeusia/ageusia (46.53%) and anosmia (30.69%). Headaches are one of the most common symptoms in many neurological pathologies such as encephalitis, meningitis, and stroke. It was shown that headaches can also occur in temporal association with a systemic viral infection [26,27,28]. Several potential pathophysiological mechanisms were suggested for headaches: A direct invasion of SARS-CoV-2 to the trigeminal nerve endings in the nasal cavity, trigemino-vascular activation due to involvement of the endothelial cells of the vessel walls with a high expression of angiotensin-converting enzyme (ACE2), and the release of the proinflammatory mediators and cytokines during COVID-19 might stimulate the perivascular trigeminal nerve endings [1,26]. Anosmia and ageusia were more frequent in the first group with the non-severe form of the disease (anosmia, 32% vs. 26.92%; ageusia, 48% vs. 42.30%; *p* < 0.05). Anosmia and ageusia were minor neurological manifestations; anosmia can arise from damage to the cells surrounding the olfactory neurons, and the olfactory tract could be a gateway for the virus to the brain. Other investigations must be carried out to verify this hypothesis and to determine if these symptoms are reversible or not [29]. 

Acute cerebrovascular events were observed in our study in 56.43% of patients, including a high proportion of ischemic stroke (50.49%). Infarctions of different arterial territories without a cardioembolic source was described in our patients. It is hypothesized that cerebrovascular manifestations of SARS-CoV-2 infection could happen as a result of the association of hypercoagulability and endothelial damage. The latter could be triggered by cytokine release, as well as viral injury, given that the endothelium also expresses ACE2 receptors [23,30]. The depletion of ACE2 by SARS-CoV-2 may cause an imbalance of the renin angiotensin system, which might result in endothelial dysfunction and ischemic stroke [20]. There was a significant difference between groups, with a higher prevalence of ischemic stroke in the second group with the severe form of COVID-19 infection (48% vs. 57.69%; *p* < 0.05).

Seizures were reported in our study as a neurological manifestation in patients infected with SARS-CoV-2 (12.87%). None of the patients were known to have epilepsy. Viral encephalitis and direct invasion of the virus to the brain may cause seizures. Our two patients (1.98%) with encephalitis presented generalized seizures and had a bad prognosis; both of them died. In addition, two cases (1.98%) were reported in connection with COVID-19 and Guillain–Barré Syndrome who presented tetraplegia, facial weakness, and sensory disturbances; they fully recovered after the specialized treatment (human immunoglobulins/plasmapheresis).

A depressed level of consciousness was reported in our study in 31.36% of patients; acute confusional syndrome or acute encephalopathy occurred in the context of infections, parenchymal damage, hypoxia, toxicity, and metabolic and electrolyte imbalances, with several underlying mechanisms. A coma at onset presented in 6.93% of the patients, all of whom had the severe form of SARS-CoV-2 infection. The patients with orotracheal intubation (5.94%) at admission to the hospital with ventilatory mechanical assistance, had the severe form of the disease and died. 

Our results indicate that patients with preexisting neurological disorders (prior stroke, 7.92%; Myasthenia Gravis, 2.97%; and Parkinson’s disease, 1.98%) and SARS-CoV-2 infection are likely to develop the severe form of COVID-19. On the contrary, these patients experienced a worsening of neurological symptoms. The most common cause of symptom exacerbation was infection, anxiety, medication errors, and delayed presentation to the hospital due to the COVID-19 pandemic [31].

Severe COVID-19 infection was significantly correlated with an increased highly sensitive C-reactive protein level (hsCRP), lactate dehydrogenase level (LDH), erythrocyte sedimentation rate (ESR), D-dimer, fibrinogen level, and blood glucose (*p* < 0.05) when compared to the first group. These biochemical parameters were increased in both groups, but the levels were much higher in the second group.

The systemic effects of SARS-CoV-2 infection might be the different mechanisms: coagulation abnormalities represented by an increase in procoagulant factors (fibrinogen, platelets, and D-dimer) and an inflammatory response with elevated levels of highly sensitive C-reactive protein (hsCRP) and erythrocyte sedimentation rate (ESR). Cardiac manifestations, arrhythmic complications, and atherosclerotic plaque rupture could be another potential mechanism assisting with the higher rate of ischemic stroke [32]. The other mechanism involves ACE2; the depletion of ACE2 by SARS-CoV-2 could cause an imbalance of the renin angiotensin system, which might result in endothelial dysfunction and ischemic stroke [33]. 

The mortality rate was 27.72% in all patients; when comparing the two groups of patients, the mortality rate was 21.33% in the first group and 46.15% in the second group with the severe form of the disease.

The limitation of our study is its retrospective nature, so selection bias may have arisen, and some important data could be missing. The recruitment of all patients from a single department and the relatively small cohort of patients are also limitations of the study. We could not determine whether the neurological disorders of the patients were caused by the SARS-CoV-2 infection or by the other factors. We wonder if neurological diseases and SARS-CoV-2 infection are concomitant or if there is a direct causal relationship.

## 5. Conclusions

The demographic, clinical, neuroradiological, and biochemical presentation of neurological disorders and concomitant COVID-19 infection in our study were similar to the conclusions provided by other studies on the same matter. Clinicians should be aware of the risk of an exacerbation of neurological symptoms in patients with SARS-CoV-2 infection. It is important that neurologists include the pathology caused by COVID-19 infection in a routine examination in order to be able to achieve the timely management of neurological manifestations and the application of specialized treatment without compromising healthcare delivery to this specific patient population. A better understanding of the potential associated neurological complications will help to reduce the impact of the COVID-19 pandemic on healthcare.

## Figures and Tables

**Table 1:** Demographic data, clinical baseline of acute respiratory syndrome SARS-CoV-2 infection, and radiological and biochemical characteristics of the study participants using the Mann–Whitney *U* test and the chi-square test.

		Total (*n* = 101)	Non-Severe Group (*n* = 75)		Severe Group (*n* = 26)		*p*-Value
No	Variable	Mean ± Std = Deviation	Mean ± Std = Deviation	Mean Rank	Mean ± Std = Deviation	Mean Rank	
		(Median)	(Median)		(Median)		
1	Age, years	70.05 ± 11.21	69.13 ± 11.64	67.76	72.69 ± 9.55	74.04	*p* < 0.05 *
2	Male, *n* (%)	63 (62.37%)	49 (65.33%)	39.24	14 (53.84%)	23.74	*p* < 0.05 *
3	Female, *n* (%)	38 (37.63%)	26 (34.67%)	21.89	12 (46.15%)	16.1	*p* < 0.05 *
	**Symptoms**						
4	Fever, *n* (%)	70 (69.30%)	46 (61.33%)	39.38	24 (92.30%)	30.6	*p* < 0.05 *
5	Dyspnea, *n* (%)	75 (74.25)	51 (68%)	43.05	24 (92.30%)	31.93	*p* < 0.05 *
6	Fatigue, *n* (%)	62 (61.38%)	40 (53.33%)	34.51	22 (84.61%)	27.47	*p* < 0.05 *
	**Blood test parameters**						
7	Leucocyte count (×1000/L)	12.85 ± 7.7	13 ± 8.27	12.45	12.42 ± 5.88	12.96	0.639
8	Lymphocyte count (×1000/L)	2.45 ± 5.41	2.78 ± 6.21	2.39	1.5 ± 1.23	1.88	0.288
9	Thrombocyte count (×1000/L)	233.53 ± 114.33	235.35 ± 118.54	226.21	228.26 ± 103.25	237.35	0.053
10	Blood glucose, mg/dL	148.05 ± 88.01	136.67 ± 61.66	142.67	180.88 ± 134.99	174.84	*p* < 0.05 *
11	LDH (U/L)	438.95 ± 482.33	356.11 ± 290.39	420.13	677.92 ± 775.23	613.8	*p* < 0.05 *
12	hsCRP, mg/L	87.88 ± 93.21	77.98 ± 95.4	84.61	116.42 ± 81.69	110.26	*p* < 0.05 *
13	VSH, mm/1 h	39.81 ± 19.8	35.76 ± 16.67	38.3	51.5 ± 23.55	48.94	*p* < 0.05 *
14	D-dimer, ng/mL	1252.09 ± 2115.51	1206.4 ± 2250.4	1185.98	1383.88 ± 1699.08	1303.1	*p* < 0.05 *
15	Fibrinogen, mg/dL	535.02 ± 189.41	521.4 ± 178.94	517.22	574.31 ± 215.78	578.39	*p* < 0.05 *
16	APTT (s)	28.04 ± 7.09	27.62 ± 5.25	27.12	29.24 ± 10.83	29.73	0.471
17	PT (s)	15.13 ± 17.79	13.17 ± 2.84	14.53	20.8 ± 34.59	19.42	0.08
18	INR	1.49 ± 1.62	1.28 ± 0.28	1.53	2.53 ± 3.69	2.27	0.421
	**Chest CT findings (pneumonia)**						
19	Unilateral, *n* (%)	33 (32.67%)	27 (36%)	21.22	6 (23.07%)	11.77	*p* < 0.05 *
20	Bilateral, *n* (%)	52 (51.48%)	32 (42.66%)	28.17	20 (76.92%)	23.82	*p* < 0.05 *
21	OTI at admission, *n* (%)	6 (5.94%)	2 (2.66%)	2.4	4 (15.38%)	3.59	0.34
22	Mortality, *n* (%)	28 (27.72%)	16 (21.33%)	14.55	12 (46.15%)	13.44	0.213

Note: * The Mann–Whitney *U* test, chi-square test, Fisher’s rest, and Z-test produced significant differences at *p* < 0.05. *n*, absolute number of patients; %, relative number of patients; LDH, lactate dehydrogenase; hsCRP, highly sensitive C-reactive protein; ESR, erythrocyte sedimentation rate; D-dimer; fibrinogen; APTT, activated partial thromboplastin time; PT, prothrombin time; INR, international normalized ratio; and OTI, orotracheal intubation.

**Table 2:** Neurological complications reported during SARS-CoV-2 infection.

No	Variable	Total, *n* = 101	Non-Severe, *n* = 75 (74.25%)	Severe, *n* = 26 (25.75%)	*p*-Value
	**Nonspecific symptoms**				
1	Headaches	73 (72.27%)	55 (73.33%)	18 (69.23%)	0.24
2	Dizziness	15 (14.85%)	9 (12%)	6 (23.07%)	0.35
	**Cerebrovascular events**				
3	Ischemic stroke	51 (50.49%)	36 (48%)	15 (57.69%)	*p* < 0.05 *
4	Cerebral hemorrhage	5 (4.95%)	2 (2.66%)	3 (11.53%)	0.08
5	Cerebral venous sinus thrombosis	2 (1.98%)	1 (1.33%)	1 (3.84%)	0.29
	**Inflammatory manifestations**				
6	Encephalitis	2 (1.98%)	0	2 (7.69%)	0.46
7	Guillain–Barré Syndrome	2 (1.98%)	0	2 (7.69%)	0.46
8	Seizures	13 (12.87%)	10 (13.33%)	3 (11.53%)	*p* < 0.05 *
	**Cranial nerve abnormalities**				
9	Anosmia	31 (30.69%)	24 (32%)	7 (26.92%)	*p* < 0.05 *
10	Dysgeusia/ageusia	47 (46.53%)	36 (48%)	11 (42.30%)	*p* < 0.05 *
11	Impaired eye movement	1 (0.99%)	1 (1.33%)	0	NA
12	Facial nerve palsy	2 (1.98%)	2 (2.66%)	0	0.38
	**Depressed level of consciousness**				
13	Acute confusional syndrome	25 (24.75%)	20 (26.66%)	5 (19.23%)	0.57
14	Coma	7 (6.93%)	0	7 (26.92%)	*p* < 0.05 *
	**Neuropsychiatric symptoms**				
15	Anxiety	24 (23.76%)	18 (24%)	6 (23.07%)	0.22
16	Depression	15 (14.85%)	11 (14.66%)	4 (15.38%)	0.33
	**Neurological comorbidities**				
17	Prior stroke	8 (7.92%)	5 (6.66%)	3 (11.53%)	0.06
18	Myasthenia Gravis	3 (2.97%)	0	3 (11.53%)	*p* < 0.05 *
19	Parkinson’s Disease	2 (1.98%)	0	2 (7.69%)	0.07

Note: * Fisher’s test and Student’s *t*-test produced significant differences at *p* < 0.05.

## Data Availability

Third-party data restrictions apply to the availability of these data. The data were obtained from Timisoara County Emergency Clinical Hospital and are available from the authors with the permission of the Institutional Ethics Committee of Clinical Studies of the Timisoara County Emergency Clinical Hospital.
